# Supplementary material for: Structure of Chlorella ohadii Photosystem II Reveals Protective Mechanisms against Environmental Stress
Source: Cells. 2023 Jul 31;12(15):1971. doi: 10.3390/cells12151971 (PMC10416949; doi:10.3390/cells12151971)
Supplement: Supplementary file 1 [file cells-12-01971-s001.zip › cells-2517127-supplementary.pdf]

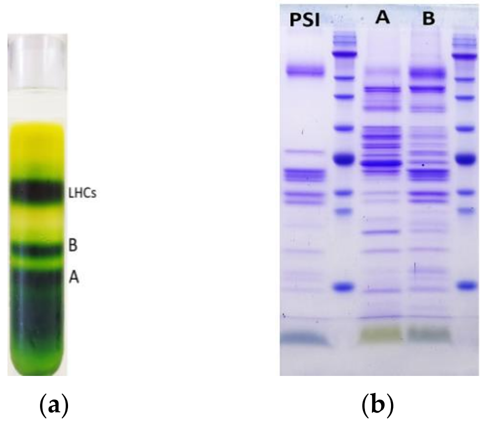

Figure S1: Sucrose gradient and SDS-PAGE of *Chlorella ohadii* PSII preparation: (**a**) Sucrose density gradient of the final *Chlorella ohadii* PSII preparation. The three fractions collected are marked A, B, and LHCs. The A fraction was used for cryo-EM data collection; (**b**) SDS-PAGE of the fractions A and B and previous PSI preparation for comparison with new preparation.

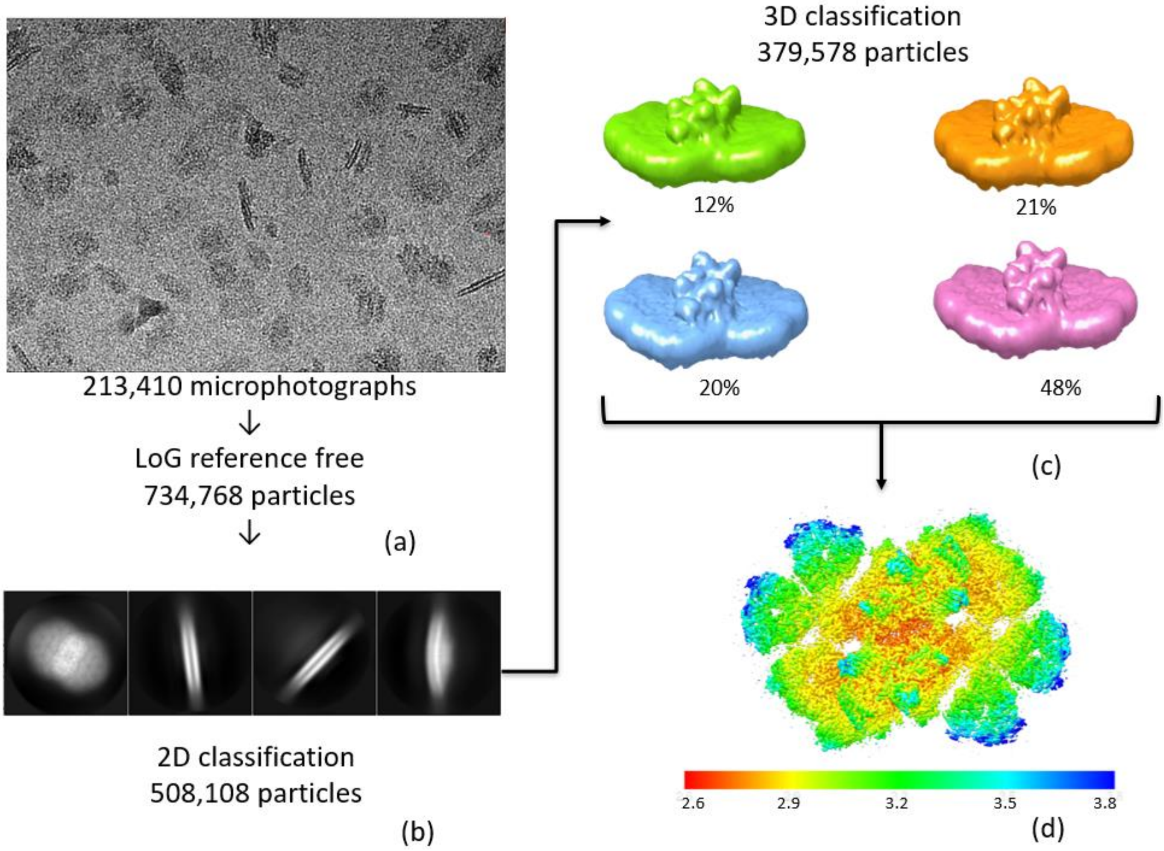

Figure S2: Cryo-EM data collection and processing scheme for unstacked PSII complex. (**a**) Sample micrograph collected for the *Chlorella ohadii* PSII dataset displaying stacked and unstacked PSII particles from multiple views; (**b**) 2D classes showing unstacked PSII complexes were created in RELION followed by 3D classification; (**c**) Chosen unstacked PSII 3D classes subjected to refinement with numbers and percentage of all chosen particles; (**d**) Final model of *Chlorella ohadii* PSII with a color-coded global resolution of 2.72 Å.

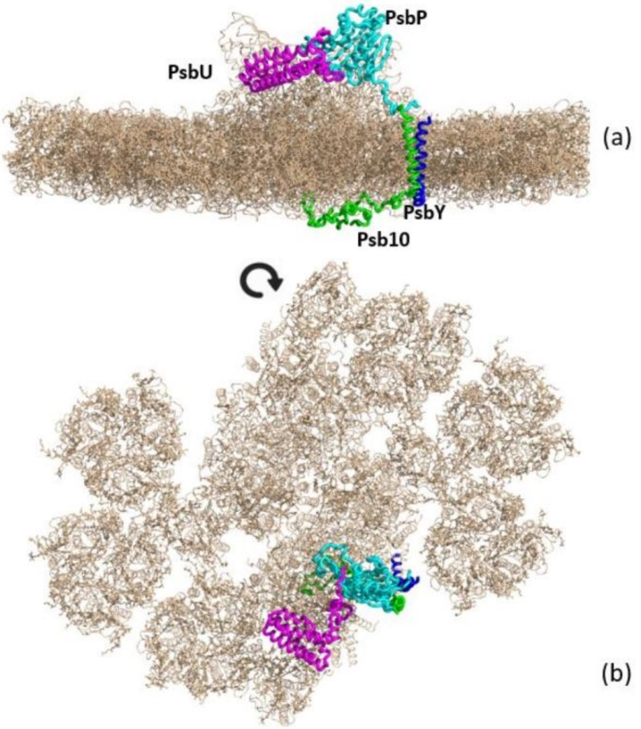

Figure S3. The added subunits are present PSII from *Chlorella ohadii* (PDB 8BD3) are shown on the background of Chlamydomonas PSII (PDB 6KAD) on which the Ohadii PSII was superimposed. PsbU is shown in magenta, PsbP is shown in cyan, PsbY is shown in blue and Psb10 is shown by green. (a) Side view along the membrane plane of PSII. (b) 90° rotation demonstrates the **t**op view of the supercomplex from the stromal side**.** Identical subunits at the same relative position are present in the second half of PSII (not shown).

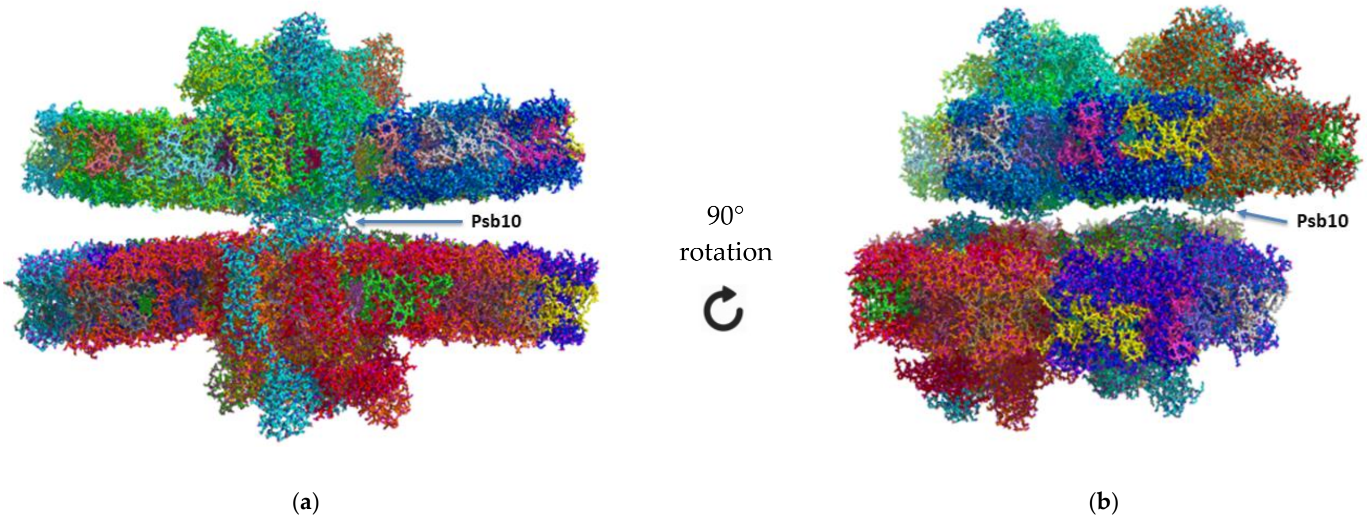

(**a**) (**b**)

Figure S4: Stacked PSII from *Chlorella ohadii* showing that Psb10 is involved in the stacking formation. (**a**) Side view along the membrane plane of stacked PSII. Psb10 may provide contacts for PSII dimer stacking; (**b**) 90° rotation in the membrane plane of the complex presented on S.Fig.3a

```
Chlorella ohadii (QFB70705.1): 4 genome repeats, 2 variants
Sbjct  160  DNRFGTIALLAVPVIGWVLFNILGPLQNQLNAMS  193
Sbjct  322  DNRFGTIALLAVPVIGWVLFNILGPLQNQLNAMS  355
Sbjct  76   DNRFGTIALLAVPVLGWVGFNILNPLQNQLDAMS  109
Sbjct  238  DNRFGTIALLAVPVLGWVGFNILNPLQNQLDAMS  271

Micractinium conductrix (PSC75791.1):5 genome repeats, 4 variants
Sbjct  144  DNRFGTIALLALPVVGWVLFNILGPLKNQIDAM  176
Sbjct  75   DNRFGTLALLAVPVVGWVGFNILGPLQNQIDAM  107
Sbjct  351  DNRFGTLALLALPALGWVGFNILGPLQNQLKSM  383
Sbjct  213  DNRFGTLALLALPALGWVGFNILGPLKNQIDAM  245
Sbjct  282  DNRFGTLALLALPALGWVGFNILGPLKNQIDAM  314

Chlamydomonas reinhardti(XP 001698338.2): 5 genome repeats, 2 variants
Sbjct  81   DNRAGILATLLVPVLGWVGFNIFGSLQAQLNQM  113
Sbjct  152  DNRVAILATLLVPVIGWVGFNIFGSLQAQLNQM  184
Sbjct  223  DNRVAILATLLVPVIGWVGFNIFGSLQAQLNQM  255
Sbjct  294  DNRVAILATLLVPVIGWVGFNIFGSLQAQLNQM  326
Sbjct  365  DNRVAILATLLVPVIGWVGFNIFGSLQAQLRQM  397

Scenedesmus sp. PABB004 (KAF8060329.1) 5 genome repeats, 3 variants
Sbjct  295  DNRFGTITLLFAPVVGWVAFNMLTPASNQLNRMN  328
Sbjct  454  DNRFGTITLLFAPVVGWVAFNMLTPASNQLNRMN  487
Sbjct  374  DNRLGTIALLFAPVVGWVGFNMLTPLFNQLNRMN  407
Sbjct  533  DNRLGTIALLFAPVVGWVGFNMLTPLFNQLNRMN  566
Sbjct  215  DNRLGTISLLFLPALGWVAFNILQPLLNQVGRMS  248

Glycine max (NP 001240940.1): 2 genome repeats, 2 variants
Sbjct  83   DNRGLALLLPIIPAIGWVLFNILQPALNQLNRM  115
Sbjct  152  DNRGQLLLFVVTPAIAWVLYNILQPALNQLNRM  184

Chenopodium quinoa (XP 021746540.1):  1genome repeat, 1variant
Sbjct  151  DNRGTLLLLVVLPAIGWVLFNILQPALNQLNKM  183
```

Figure S5. Number of different copies of the PsbY, presented in the single transcripts of 4 algae and 2 high plants.

In algae and other lower plant species represented in the Protein Data Bank (PDB), it is observed that there are typically 3-5 copies of the PsbY subunit present in their Photosystem II (PSII) structures. In contrast, higher plants predominantly exhibit 1 or occasionally 2 copies of the PsbY subunit in their PSII complexes.
